# Health Needs and Access to Healthcare Services in Migrant Populations in Greece: Data From the Hprolipsis Study

**DOI:** 10.7759/cureus.78196

**Published:** 2025-01-29

**Authors:** Olga Anagnostou, Natassa Kalpourtzi, Argiro Karakosta, Agis Terzidis, Anastasios Yfantis, Alexios Margalias, Revekka Tzanetea, Eleftherios Pallis, Magda Gavana, Apostolos Vantarakis, Grigoris Chlouverakis, Giota Touloumi

**Affiliations:** 1 Department of Hygiene, Epidemiology and Medical Statistics, National and Kapodistrian University of Athens School of Medicine, Athens, GRC; 2 Department of Social Work, University of West Attica, Athens, GRC; 3 Department of International Medicine - Health Crisis Management, National and Kapodistrian University of Athens School of Medicine, Athens, GRC; 4 Polyclinic, Doctors of the World, Greek Delegation, Athens, GRC; 5 Polyclinic, Programs of Development Social Support and Medical Cooperation (PRAKSIS), Athens, GRC; 6 Department of Primary Health Care, General Practice and Health Services Research, Aristotle University Medical School, Thessaloniki, GRC; 7 Department of Public Health, Medical School, University of Patras, Patras, GRC; 8 Laboratory of Biostatistics, University of Crete School of Medicine, Heraklion, GRC

**Keywords:** emeno study, greece, hbv, hcv, healthcare access, hiv, hprolipsis, migrants

## Abstract

Introduction

Migration to Greece continues with a constant influx of immigrants from several countries. The importance and necessity of data related to migrants’ health and needs are widely recognized, yet scarce and sporadic. This analysis aims to present data on migrants’ health needs and to investigate potential barriers to meeting them.

Methods

Data were collected during 2013-2016 in the framework of the Hprolipsis study, a health survey in Greece designed to study HIV, hepatitis B virus (HBV), and hepatitis C virus (HCV) prevalences in the general population, Roma, and immigrants. Migrants over 18 who had lived in Greece for at least six months were interviewed and, after consent, filled in a specially designed standardized questionnaire. Multivariable logistic models were applied to identify factors associated with unmet health needs.

Results

In total, 612 migrants participated in the study, (55.6% male, age median 36.9 (interquartile range (IQR) 29.8, 46.4) years, and median stay time 96 (IQR 36.0, 180.0) months. The majority (63.1%) rated their health status as good/very good while 28.3 % reported suffering from a chronic disease. The majority (87.3%) reported health needs during the last 12 months, and a quarter (25.9%) declared unable to meet them. Chronic diseases (adjusted odds ratio (OR): 1.71; 95%CI 1.03, 2.83), poor/very poor health (OR:2.49; (95%CI 1.29, 4.82)), and moderate or severe food insecurity (OR:2.39; 95%CI 1.38, 4.14 and OR 4.03; 95%CI 1.97,8.23, respectively) were identified as aggravating factors for unmet needs.

The main reason for unsatisfied health needs was service cost (69.5%) with personal, migrant-status, and social-related reasons representing lower prevalence (27.5%, 21.4%, and 14.5%, respectively). Among those with unmet health needs, age ≥ 40 years (OR=2.66; 95%CI 1.01, 6.97) and unemployment (OR=3.39; 95%CI 1.30, 8.84) were significantly associated with higher odds of reporting economic difficulties while Asian and African origin with significantly lower odds compared to those from the Balkan territory (OR 0.21; 95%CI 0.07, 0.69 and OR 0.25; 95%CI 0.08, 0.82, respectively).

Conclusions

The more vulnerable migrants (poor/very poor health, chronic disease, and food insecurity) are more likely to be unable to meet their health needs. Health services cost is the most prevalent reason for unmet needs while older age and unemployment increase financial barriers. Expanding access to healthcare for all migrants is required to meet their unmet health needs, in addition to preventive measures aiming to cover basic needs such as food provision, vaccination, and socioeconomic support.

## Introduction

Migration is an intertemporal social phenomenon in global human history, rooted in the perpetual effort of humankind to feel safe and to improve its living conditions. The number of international migrants was estimated to be 281 million in 2020 (3.6% of the world’s population), with Europe hosting, relatively to its size, the largest number (82.3 million) [1,2]. Qualitative and quantitative characteristics of the phenomenon are associated with specific social conditions in the country of origin (e.g. wars and all kinds of armed conflicts, economic crises, and poor living conditions). In turn, migration affects the transit and the final destination countries in several ways and varying degrees [3-6].

Many researchers dispute the once popular "healthy migrant effect," at least in terms of its strength, time stability, and universality [7-11]. This effect suggests that migrants in high-income countries exhibit lower mortality rates than the native population, a phenomenon often attributed to the positive health selection of individuals who migrate for work or educational opportunities. However, this initial health advantage may diminish as migrants face socioeconomic and environmental challenges in host countries. Undoubtedly, one of the challenges for host countries is the capability of their national health systems to efficiently address the needs of both the general and the migrant populations effectively, underscoring the role of human mobility as a critical factor in public health [12-15]. Despite its frequent citation, limited data constrain a full understanding of the health outcomes of migrants, especially for marginalized groups in low- and middle-income settings. Thus, researchers stress the importance of investigating additional factors and considering the varied experiences of migrant populations to contextualize the healthy migrant effect better [11].

Knowledge of the actual health needs of a given population and identification of potential barriers to covering them are indisputable prerequisites for designing targeted actions at the public health level. For migrants, data on existing "objective" (i.e., at the national health system level) and "subjective" barriers (i.e., at a personal level in the sense that migrants themselves perceive them as such, including cultural differences, linguistic restrictions, ignorance of the administrative procedures and their rights, experience or fears of possible racist behaviors) are limited. However, knowing the barriers and their potential association with other socioeconomic factors is essential for public health situation analysis and for adopting effective policies, addressing both, “objective” and “subjective” barriers.

Greece, a traditional migrants’ departure country until the 1970s, has become a host country for the last three decades. The first immigration waves, in the early 1990s, were related to the disintegration of the former Eastern bloc countries. As a result, most immigrants at that time came from neighboring countries (e.g., Albania) or countries of the former Union of Soviet Socialist Republics (USSR) (e.g., Georgia). The second wave of migration to Greece, mainly a result of instability in the Arab countries of Africa and Asia, continues to this day. Large numbers of immigrants, even when Greece is not their final destination, are forced to remain in the country for an indefinite amount of time, under conditions that raise serious questions regarding access to the health care system [16].

Following the recent economic crisis, several scientific articles from Greece have reported its effects on people’s health and the country's health system [17,18]. Being a migrant seems to deteriorate access to health and unmet pharmaceutical needs compared to the general population [19]. However, data are scarce and sporadic, although their importance and necessity are widely recognized.

The Hprolipsis study is the first health survey in Greece designed to study the prevalence of HIV, hepatitis B virus (HBV), and hepatitis C virus (HCV) in the general population, Roma, and immigrants in Greece [20]. Among other health determinants related to HIV, HBV, and HCV, data on health status self-assessment, actual health needs during the last 12 months, and needs satisfaction, along with the main reasons that prevented them, were collected. 

The aim of the study was to use data from the Hprolipsis study to describe the access to health services of the migrant population in Greece from 2013 to 2016, their unmet health needs, and the main potential barriers, and to investigate the factors associated with these.

## Materials and methods

This was a study based on data from the Hprolipsis survey, which was conducted by the National and Kapodistrian University of Athens Medical School in collaboration with all the other medical schools in Greece and the non-governmental organizations, Doctors of the World and Programs of Development Social Support and Medical Cooperation (PRAKSIS) [20]. The Hprolipsis study started in May 2013 and was completed in June 2016. Ethics Committee of the National and Kapodistrian University of Athens issued approval 6141, dated March 4, 2015.

Study participants

For this study, migrants were defined as those born in another country. Inclusion criteria were (i) aged ≥18 years at recruitment and (2) living in Greece for at least six months (self-reported); thus, new arrivals and/or those living in shelter camps were excluded. Those from the EU-15 countries (15 member states of the European Union (EU) as of December 31, 2003), Norway, Switzerland, the United States, and Canada were also excluded.

Hprolipsis study

The Hprolipsis study was implemented in the framework of the action “Design and Development of a Viral B and C Virus and HIV Infection Control Program in the General Population and in Displaced Populations”, which was co-funded by the European Community (European Social Fund) and national resources [19].

Hprolipsis was carried out in synergy with the National Survey of Morbidity and Risk Factors (EMENO) study, a health examination survey of the general adult population living in Greece to investigate population morbidity. The EMENO study focused on cardiovascular and respiratory diseases and their associated risk factors [21]. EMENO’s target sample was 6000 persons.

The lack of reliable data on the exact composition of migrants as well as the high mobility of the population during the study period made it very difficult to select a random sample. In the lack of an appropriate sampling frame, a two-step procedure was followed [19]. Briefly, based on the 2011 census, the percentage of those born abroad was around 10% of the total people living in Greece, with more than half of them originating from Albania; to keep the sampling fraction similar to that in the general population sample in the EMENO study, the migrants target sample was set to 600 [21]. Albanians migrated to Greece in the late 90s to early 2000s and most of them are currently well integrated into Greek society. As this survey focused on the most vulnerable populations, it was a priori decided, in our sample, to underrepresent those originating from Albania and instead to overrepresent migrants who constitute the more recent wave of migration, namely those originating from Africa and Asia.

In the absence of reliable data on the reference population, it has been suggested to approach migrants through multiple sources in order to reduce selection bias [22]. To implement the migrants’ survey, a network of NGOs and migrant communities was set up, and the survey’s aims were disseminated to them. More specifically, an official collaboration with the two largest NGOs serving migrants in Greece was set up: The Greek delegation of Doctors of the World and the NGO PRAKSIS (Programs of Development Social Support and Medical Cooperation). These NGOs run open polyclinics in four large cities/regions in Greece where the majority of migrants live: Athens-Attica (Doctors of the World and PRAKSIS), Patra-Peloponnese, Western Greece (Doctors of the World), Thessaloniki-Central Macedonia, Northern Greece (Doctors of the World and PRAKSIS) and Chania-Crete (Doctors of the World). Eligible migrants attending the polyclinics were invited to participate in the study. As those attending NGOs’ polyclinics may have differed from the general migration population, eligible migrants were also invited through their communities to visit polyclinics on specific days and times.

Study Procedures and Data

The Hprolipsis Steering Committee, consisting of experts in epidemiology, medical statistics, virology, internal medicine, and hepatology and representatives of the collaborating NGOs, migrants, and Greek Roma communities, developed generic questionnaires using items previously validated whenever possible [20,21,23]. Specific modifications were made to the migrants’ questionnaire to facilitate communication and to include specific migrant questions such as on living conditions and legal status.

The survey questionnaire of the Hprolipsis study was tested and validated (time, clarity of questions, comprehensibility, etc.) in four migrant volunteers from each cooperating NGO. Feedback from questionnaire testing led to minor modifications/corrections where necessary. A specially trained interviewer interviewed the participants who had been informed and signed the consent form (available in six languages: Greek, English, French, Russian, Albanian, and Arabic). The main thematic units of the study questionnaire were: demographic/ socioeconomic status; self-reported general health status and health services use, unmet needs and health barriers factors affecting health; living conditions, and blood-transmitted infectious diseases: HIV, HBV, and HCV.

In the current study, we focused on the first three thematic units.

Statistical analysis

Health status (very good, good, moderate, poor, and very poor) as well as the existence of chronic diseases (yes/no) was self-reported. Actual health need was defined as any health need during the last 12 months (yes/no); among those declaring having health needs, whether their needs were satisfied (yes/no) and if not for which reason(s). Participants could provide more than one reason/barrier for unmet health needs. The reasons for the inability to cover health needs were grouped into five groups: economic, social, migrant, personal, and other. The relevant parts of the questionnaire and the grouping are presented in the supplementary materials.

Means and standard deviation (SD) or medians and interquartile range (IQR) were used to describe continuous variables and percentages for categorical variables, as appropriate. The Chi-square test was used to assess for differences. Multivariable logistic regression models were fitted to assess the factors associated with the probability of having unmet health needs and for specific reasons of unmet health needs. All analyses were performed using the Stata Statistical Software: Release 13 (2013; StataCorp LLC, College Station, Texas, United States).

## Results

In total, 612 migrants participated in the study, the majority of them being male (55.6%), with a median age of 36.9 (interquartile range (IQR) 29.8, 46.4) years. Their median time of stay in Greece was 96 (IQR 36.0, 180.0) months. The majority (n=520, 84.9%) received less than 12 years of education, were unemployed (59%), had no insurance coverage (84.6%), and had very low income (58.7% reported a personal income of less than 350 euros per month). The characteristics of the study population are presented in Table 1.

**Table 1:** Descriptive characteristics of the Hprolipsis study migrant population (N=612) ^*1^ One person from Honduras USSR: Union of Soviet Socialist Republics

Characteristics	Frequency	Percentage
Gender		
Male	340	55.6
Female	272	44.4
Age group (years)		
18-29	156	25.5
30-39	211	34.5
40-49	130	21.2
50-59	80	13.1
>60	35	5.7
Country of origin		
Asia - Middle East^*1^	186	30.4
Africa	145	23.7
Albania	159	26.0
Rest Balkan territory	62	10.1
ex-USSR countries - East Europe	60	9.8
Years of education		
>12	92	15.0
>9 to ≤12	242	39.5
>6 to ≤9	164	26.8
0-≤6	114	18.6
Marital status		
Single	214	35.0
Married	331	54.1
Divorced/ Widowed	65	10.6
No answer	2	0.3
Staying with partner		
Yes	268	43.8
No	337	55.1
No answer	7	1.1
Have children		
Yes	405	66.2
No	206	33.7
No answer	1	0.2
Members living in a household, median (IQR)	3 (2, 5)	
Housing type		
Homeless (long-term)	4	0.7
Occasionally homeless	16	3.3
Hosted in a shelter/apartment/hotel	57	12.6
Owned residence	48	20.4
Rented residence	374	81.5
Staying with friends/family	99	16.2
Unknown	14	2.3
Household surface (m^2^), median (IQR)	50 (35, 65)	
Food insecurity		
No	443	74.0
Moderate	104	17.4
Severe	52	8.7
Personal Income (Euros per month)		
≤350	359	58.7
351 – 700	96	15.7
>700	20	3.3
No answer- I don’t know	137	22.4
Employment status		
Unemployed	361	59.0
Employed	160	26.1
Retired	64	10.5
Student	5	0.8
Disabled	7	1.1
Housekeeping	5	0.8
Unknown	10	1.6
Insurance coverage		
Yes	83	13.7
No	518	84.6
I don’t know/Unknown/No answer	8/2/1	1.8
Legal status		
With documents	312	51.0
Asylum seeker	150	24.5
Without documents/unable to specify	149	24.4
Unknown	1	0.2

Most participants (n=382, 63.1%) rated their health status as good/very good, with 159 (26.3%) rating it as moderate, 64 (10.5%) as poor/very poor, and 163 (28.3%) reported suffering from a chronic disease. Eighty-one (13.2%) individuals reported hospitalization needs during the last 12 months, while 198 (32.3%) visited a public hospital and 296 (48.4%) visited an NGO health service. The majority (522/612, 87.3%) reported having any health needs during the last 12 months. The multivariable analysis (data not shown) revealed that origin from the African region (OR 0.34, 95%CI: 0.18-0.65) was the only factor significantly associated with lower odds of reporting health needs compared to other origins.

Of the 522 who reported health needs, 131 (25.9%) declared unable to meet them. Factors associated independently with unmet health needs are presented in Table 2. Migrants having kids were less likely to report unmet health needs (OR: 0.46, 95%CI: 0.21-0.99). On the contrary, those suffering from chronic diseases (OR: 1.71, 95%CI: 1.03 to 2.83), those evaluating their health as poor/very poor (OR: 2.49, 95%CI: 1.29-4.82), and those reporting moderate or severe food insecurity (OR: 2.39, 95%CI: 1.38- 4.14 and OR: 4.03 95% CI: 1.97-8.23, respectively) were significantly more likely to report unsatisfied health needs.

**Table 2:** Factors associated with unmet health needs during the last 12 months: results from multivariable logistic regression (N=475)** * Baseline category ** 47 of the 522 migrants who reported having health needs were excluded from the multivariable model because of missing data in one or more of the model covariates.

Covariate	Odds Ratio	95% CI	P>|z|
Gender			
Women*	1		
Men	0.684	(0.423,1.108)	0.123
Age			
<40 years*	1		
≥40 years	0.977	(0.576,1.657)	0.932
Family status			
Not married*	1		
Married	1.787	(0.822,3.882)	0.143
Divorced/widowed	2.335	(0.918,5.935)	0.075
Health status evaluation			
Moderate/Good/Very good*	1		
Poor/Very poor	2.492	(1.289,4.820)	0.007
Chronic disease			
No*	1		
Yes	1.706	(1.030,2.825)	0.038
Having Kids			
No*	1		
Yes	0.457	(0.212,0.987)	0.046
Food insecurity			
No*	1		
Moderate	2.390	(1.380,4.139)	0.002
Severe	4.027	(1.970,8.229)	<0.001

**Figure 1:**
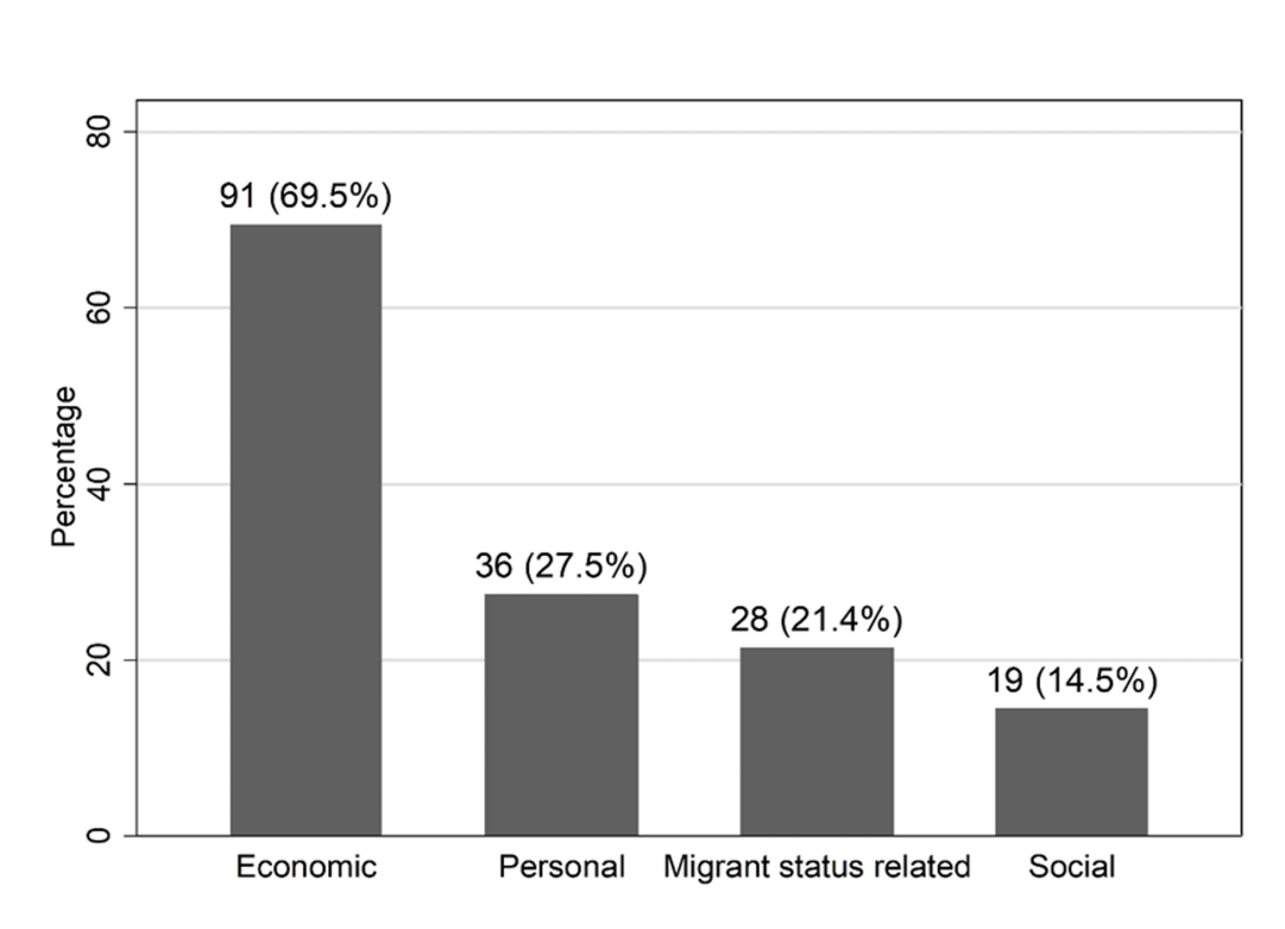
Reasons for not being able to access health care services when needed (N=131)

The prevalence of the grouped reported healthcare access barriers is presented in Figure 1 whereas reported frequencies of each itemized reason are presented in the Appendices. A total of 131 participants had reported an inability to meet health needs. Of these 131, difficulty in covering the cost of health services was the most frequently reported reason (n=91, 69.5%), followed by personal (n=36, 27.5%), migrants status-related (n=28, 21.4%), and social (n=19, 14.5%) reasons. Among personal reasons, the most prevalent item was “I don’t know a good doctor or specialist”. The most frequent choices among migrant and social reasons were those related to structural barriers of the health system (“I don’t speak Greek”, “I don’t understand how the system works” for migrant related and “difficult to make an appointment”, “long waiting list” for social reasons). 

Reported reasons for unmet needs by several personal characteristics are presented in Table 3. Among those with unmet health needs, men, those aged ≥40 years, living in Greece for more than eight years, having kids, and coming from Albania, remaining Balkan countries, or ex-USSR/East Europe countries had higher percentages of reporting cost as one of the reasons of unmet health needs. Interestingly, asylum seekers reported costs less frequently (46.4% vs 78.3% for those with legal documents and 69.7% for those without documents).

**Table 3:** Reported reasons for unmet health needs by persons’ characteristics (N=131) *p-values were obtained using chi-squared test

	Financial Reason	p-value*	Migrant-related Reason	p-value*	Social Reason	p-value*	Personal Reason	p-value*
	Frequency (Percentage)		Frequency (Percentage		Frequency (Percentage		Frequency (Percentage	
Total	91 (69.5)		28 (21.4)		19 (14.5)		36 (27.5)	
Gender		0.024		0.025		0.322		0.684
Male (n=62)	49 (79.0)		8 (12.9)		7 (11.3)		16 (25.8)	
Female (n=69)	42 (60.9)		20 (29.0)		12 (17.4)		20 (29.0)	
Age group (years)		0.010		0.023		0.523		0.055
18-29 (n=35)	20 (57.1)		10 (28.6)		6 (17.1)		14 (40.0)	
30-39 (n=39)	23 (59.0)		13 (33.3)		7 (17.9)		12 (30.8)	
≥40 (n=57)	48 (84.2)		5 (8.8)		6 (10.5)		10 (17.5)	
Staying with partner		0.390		0.558		0.825		0.526
Yes (n=85)	61 (71.8)		17 (20.0)		12 (14.1)		22 (25.9)	
No (n=45)	29 (64.4)		11 (24.4)		7 (15.6)		14 (31.1)	
Personal Income (Euros per month)		0.095		0.124		0.838		0.108
<350 (n=77)	55 (71.4)		21 (27.3)		11 (14.3)		25 (32.5)	
351-700 (n=19)	15 (78.9)		1 (5.3)		3 (15.8)		4 (21.1)	
>700 (n=4)	1 (25.0)		1 (25.0)		1 (25.0)		3 (75.0)	
Educational level		0.018		0.040		0.305		0.090
Up to primary (n=33)	17 (51.5)		12 (36.4)		7 (21.2)		13 (39.4)	
Secondary /post-secondary (n=78)	61 (78.2)		10 (12.8)		10 (12.8)		15 (19.2)	
Higher than secondary (n=15)	11 (73.3)		4 (26.7)		1 (6.7)		6 (40.0)	
Employment Status		0.236		0.300		0.854		0.220
Employed (n=31)	19 (61.3)		5 (16.1)		4 (12.9)		8 (25.8)	
Unemployed (n=79)	60 (75.9)		21 (26.6)		13 (16.5)		18 (22.8)	
Retired/ Household (n=16)	10 (62.5)		2 (12.5)		2 (12.5)		7 (43.8)	
Years living in Greece		0.011		0.001		0.371		<0.001
≤8 years (n=62)	36 (58.1)		21 (33.9)		11 (17.7)		27 (43.5)	
>8 years (n=66)	52 (78.8)		7 (10.6)		8 (12.1)		9 (13.6)	
Having Kids		0.003		0.041		0.410		0.452
No (n=44)	23 (52.3)		14 (31.8)		8 (18.2)		14 (31.8)	
Yes (n=86)	67 (77.9)		14 (16.3)		11 (12.8)		22 (25.6)	
Insurance		0.340		0.255		0.058		0.898
No (n=116)	83 (71.6)		26 (22.4)		15 (12.9)		31 (26.7)	
Yes (n=12)	7 (58.3)		1 (8.3)		4 (33.3)		3 (25.0)	
Chronic disease		0.226		0.954		0.562		0.989
No (n=72)	46 (63.9)		15 (20.8)		12 (16.7)		20 (27.8)	
Yes (n=47)	35 (74.5)		10 (21.3)		6 (12.8)		13 (27.7)	
Health status evaluation		0.434		0.054		0.650		0.571
Moderate/Good/Very good (n=101)	69 (68.3)		18 (17.8)		14 (13.9)		26 (25.7)	
Bad/Very bad (n=29)	22 (75.9)		10 (34.5)		5 (17.2)		9 (31.0)	
Country of origin		<0.001		0.001		0.117		0.018
Albania (n=37)	32 (86.5)		1 (2.7)		5 (13.5)		5 (13.5)	
Rest Balkan region (n=18)	14 (77.8)		2 (11.1)		0 (0.0)		4 (22.2)	
Ex USSR- East Europe (n=15)	14 (93.3)		3 (20.0)		1 (6.7)		2 (13.3)	
Asia- Middle East (n=35)	16 (45.7)		11 (31.4)		9 (25.7)		16 (45.7)	
Africa (n=26)	15 (57.7)		11 (42.3)		4 (15.4)		9 (43.6)	
Legal status		0.009		0.001		0.430		0.008
With documents (n=69)	54 (78.3)		6 (8.7)		7 (10.1)		13 (18.8)	
Seeking asylum (n=18)	13 (46.4)		11 (39.3)		5 (17.9)		14 (50.0)	
Without documents (n=33)	23 (69.7)		11 (33.3)		6 (18.2)		9 (27.3)	

Migration-related reasons were reported more frequently in women compared to men, in those aged <40 years, with a level of education up to primary, living in Greece for less than eight years, not having kids, and in those coming from Africa and Asia/Middle East compared to Balkan or ex-USSR/East Europe countries. Legalized immigrants report significantly lower rates of immigration-related factors as reasons for not meeting health needs (8.7% vs 39.3% for asylum seekers and 33.3% for those without documents). Personal reasons were reported more frequently in those living in Greece for less than eight years, those from Africa and Asia/Middle East, and those seeking asylum (50.0% vs 18.8% for those with documents and 27.3% for those without documents). For social reasons, although no significant gender difference was observed, younger individuals (i.e., aged 18-49) were more likely to cite social barriers compared to older participants. Additionally, those from Africa and Asia/Middle East were more likely to report social reasons than participants from the Balkans or former USSR/Eastern Europe, though this difference did not reach statistical significance.

A multivariable logistic regression among those reporting unmet health needs (N=131) was fitted to identify factors independently associated with reporting economic barriers to meeting health needs. The results are presented in Table 4. Among those with unmet health needs, older people (≥40 years compared to <40 years, OR=2.66; p-value=0.047) and the unemployed (compared to employed, OR=3.39; P-value=0.013), had significantly higher odds of reporting economic difficulties in meeting health needs. On the other hand, migrants with origin from Asian-Middle East and African countries had significantly lower odds of reporting cost-related problems compared to those from Balkan territory (OR=0.21; p=0.01 and OR=0.25, p=0.022, respectively).

**Table 4:** Factors associated with cost as a reason for unmet health needs during the last 12 months: Results from multivariable logistic regression (cost versus other reason) (N=131) * Baseline category USSR: Union of Soviet Socialist Republics

Covariate	Odds Ratio	95% CI	P>|z|
Gender			
Female*	1		
Male	0.574	(0.205, 1.605)	0.290
Age			
<40 years*	1		
≥40 years	2.655	(1.012, 6.965)	0.047
Unemployed			
No*	1		
Yes	3.387	(1.300, 8.841)	0.013
Region of origin			
Balkan territory*	1		
Ex-USSR- East Europe	1.478	(0.157, 13.864)	0.733
Asia- Middle East	0.211	(0.065, 0.684)	0.010
Africa	0.252	(0.078 to 0.821)	0.022

Seeking asylum and lacking legal documents dramatically increase the odds of presenting unmet health needs due to immigration-related factors (OR=5.842; p=0.005 and OR=5.94; p=0.005 respectively), after adjusting for gender, age, and employment status (data not shown).

## Discussion

In a large sample of immigrants in Greece, we found that a high percentage reported health needs and a significant proportion declared unable to meet them, primarily due to cost reasons. Most migrants evaluated their health as good/very good; however, the proportion that rated their health status as poor/very poor (10.6%), is, even if slightly, higher than that of the general population in the EMENO study (6.0%) (unpublished data from Touloumi et al.'s ongoing study [21] via personal communication, 2025). At the same time, chronic conditions were reported by 26.6%, a rate much lower than that in the general population in the EMENO study (50.1%), indicating either different perceptions of health status or reflecting the insecure feeling of the migrant population compared to the general population (unpublished data from Touloumi et al.'s ongoing study [21] via personal communication, 2025). Despite the low rates of having chronic diseases, almost nine out of 10 in our study reported health needs during the last year.

While other studies conducted in Greece have investigated the health needs of the immigrant population [18,24-26], to our knowledge, the current study has the highest reported percentage of health needs by migrants (87.3%), possibly related to the period of the study implementation which was the peak of migrant influx in Greece. On the other hand, 32.3% of study migrants used any health service, and 13.2% needed hospitalization; percentages similar to those previously reported (49.7% of migrants used health services during the last year, and 13.1% were hospitalized) [24].

From a public health perspective, the most disturbing finding is not existing health needs but the inability to meet them. In this study, 25.9% of migrants reported unmet healthcare needs; based on the EMENO study (unpublished data from Touloumi et al.'s ongoing study [21] via personal communication, 2025), the corresponding percentage for the general population was 21.9% indicating similar rates of unmet health needs in migrants and the general population. As in our study, the MIGHEAL study, conducted in the same period (that is, in the middle of the serious financial crisis in Greece) found that, contrary to other European countries, there was no significant difference between migrants and non-migrants in Greece, even though migrants report slightly higher percentages of unmet health needs [25]. A more recent study though, comparing the unmet needs in four European countries using the European Union Statistics on Income and Living Conditions (SILC) data from 2019, showed that immigrants living in Greece present the uppermost probability of unmet needs (39.3%) compared to those living in Germany (4.1%) [27]. Combined, these results show that in periods of financial crisis, the general population and migrants may have similarly high rates of unmet health needs. Still, out of crisis periods, migrant populations tend to have higher rates of unmet health needs. In any case, the finding that one in four migrants declares unmet health needs is an alarming sign from public health and human rights perspectives.

More alarming, among migrants, those with chronic health problems, those who rate their health as poor/very poor, and those experiencing food insecurity present a significantly higher probability of being unable to meet their health needs. It seems like the greater need for health services multiplies the difficulty in covering those needs. Our finding agrees with another study from Greece which identified poor health per se as a prognostic factor for unmet needs in a mixed migrant and non-migrant population [19]. A recently published article using European Health Interview Survey data from 2013-2015 covering 27 countries and 12,817 migrants found a similar overall prevalence of unmet healthcare needs among migrants in Greece (27.8%) and confirms the aggravating role of low-income, and poor health on having unmet health needs [28].

Surprisingly, the only protective factor for unmet health needs was having kids. A possible explanation for this finding is that parenthood is a surrogate marker of being a member of a social network. Having a social network had significantly lower levels of unmet needs in the previously mentioned study [28]. Nevertheless, among those with unmet needs those having kids report more frequently financial barriers to cover them, indicating that as the number of family members grows, so does the financial burden.

The most frequently reported reason for unmet health needs was the inability to cover the cost (69.5%), while reasons related to migrant status, and personal or social reasons followed at a great distance. In the study of Galanis et al., high cost as a reason was reported in lower percentages (34.5%), followed by long waiting times in hospitals (12.6%) and lack of free time (9.2%) [24]. It has to be mentioned that all participants in their study were documented immigrants, with significantly higher incomes and lower unemployment rates compared to our study sample, possibly because the Greek financial crisis had not yet been declared. A recent review of unmet healthcare needs in Southeastern Europe identified the high cost of services as the main barrier to health services access in the region [29].

Age neither alters the frequency of reporting health needs nor the ability to cover them in our sample of migrants; however, among those with unmet health needs, younger individuals (>40 years) present a higher prevalence of reporting migrant-related barriers. Nevertheless, in the multivariate analysis, age ≥40 increases the odds of economic obstacles to meeting health needs (OR 2,65, p 0.047). In other words, when older people report access problems, it is mainly due to economic reasons rather than immigration, social, or personal ones.

In our multivariate analysis, low income was not a statistically significant aggravating factor for financial barriers. A possible explanation is that only four of the 131 persons with cost-related barriers reported income ≥700 euros, which was the lower official salary in Greece at that period. Thus, there was not much variability in participants’ income to allow the detection of a potentially significant association. However, completely expected and understandable, unemployment triples the likelihood of reporting cost-related barriers to health services. Unemployment rates were very high among our study population (59%) while at the same period, the rate for the general Greek population was approximately half (25%) [30]. The harmful effects of unemployment on health are recognized widely [31], although the various pathways in which these effects occur need specification. What is certain and beyond doubt is that those who are most at risk of poor health are at a higher risk of being unable to afford the cost of health services.

It is noteworthy that women, younger individuals, those with lower than primary education, those living in Greece for less than eight years, those not having kids, asylum seekers, and undocumented migrants, present structural barriers more frequently than financial ones. More interesting is the pattern observed concerning the relation between migrants' origin and unmet health needs. Migrants originating from Africa presented higher percentages of migrant-related and personal barriers to meeting their health needs. Additionally, the only protective factor for reporting economic barriers to health needs was the origin from African (OR 0.252, p 0.022) and Asian (OR 0.211, p 0.010) countries. It has to be mentioned that by its design, the questionnaire allowed multiple choices in answering reasons for unmet health needs. The respondents, although not obliged to do it, may have felt the subjective need to prioritize their problems. As a consequence, an inaccurate analysis could lead to the erroneous conclusion that migrants from Africa and Asia are in better condition because they report less frequent financial barriers. The truth may be just the opposite: these immigrants may face severe structural barriers as they more often report that they “do not have legal documents”, “don’t understand how the system works or they don’t know if they have the right”, “don’t speak the language”, and “are afraid they would be arrested or deported”.

A similar paradox is observed regarding the legal status. Asylum seekers present significantly lower rates of stating cost as a reason for non-access compared with the rest of immigrants with or without documents (46.4% vs 78.3% and 69.7%, respectively, p= 0.009). Furthermore, in the multivariate analysis, asylum seekers and undocumented migrants have almost six times higher odds of presenting unmet health needs due to immigration-related factors (data not shown). The finding should be related to the prioritization of needs (which may depend on many personal and social confounders) and not on an assumed economic strength of the particular immigrant group. We must also mention that asylum seekers in Greece are covered by the National Health System at least until the final response to their request; however, structural barriers still exist and hinder the satisfaction of health needs [32-34]. 

The strengths of our study are the large sample size and the extended questionnaire which offers the opportunity to study several aspects of the health access issue. Addressing migrants as a homogenous group is the rule in some studies on migrant healthcare use [35]. Our migrant sample, although not random, was scheduled to permit comparisons of several subgroups among migrants.

Despite the significant advantages of the study, it also had several limitations. The lack of data on the exact composition of the migrant population in Greece forced us to use a convenient sample. Other convenient methods have been used by other researchers in similar cases [19,36]. Some researchers applied the same sampling methods to the migrant and the general populations [25]. By definition, in our sample, recent entrants (<6 months), regardless of their nationality, were excluded while immigrants from Albania were under-represented as they are considered, not unjustifiably, almost fully integrated into the general population; in a study of 1152 migrants during 2013-2014, 93.2% of Albanians possessed legal documents and 77.7% valid insurance [35].

Immigrants' health needs and their access to health services represent major public health issues worldwide [36]. Further research on migrants’ health issues is an undoubtful priority to understand the population's needs, detect gaps, and identify obstacles [37]. Access to health services for migrants is a prerequisite for the successful integration of migrants in the country of destination [38,39]. Expanding access to healthcare for all migrants to meet their unmet health needs, along with a full and continuous covering of their nutritional needs, implementation of preventive measures, psychosocial support, and acceleration of procedures for asylum seekers represent the only way to guarantee their security and respect of human rights.

## Conclusions

The more vulnerable migrants (poor/very poor health, chronic disease, and food insecurity) are more likely to be unable to meet their health needs. Health services cost is the most prevalent reason for unmet needs while older age and unemployment increase financial barriers. Expanding access to healthcare for all migrants is required to meet their unmet health needs and guarantee their human rights.

## Appendices

**Table 5:** Itemized questions of the reasons for unmet health needs in migrants in Greece (N=131): Classification of reasons and frequency

The precise wording of asked questions	Classification	N	%
“I had trouble paying the costs”	Economic reason	91	69.5
“I found it difficult to make an appointment”	Social reasons	12	9.2
“I don’t speak the language and I cannot understand”	Migrant reasons	11	8.4
“I don’t understand how the system works and I do not know if I have the right”	Migrant reasons	11	8.4
“I don't have the legal documents”	Migrant reasons	9	6.9
“I went but I was denied medical coverage”	Migrant reasons	9	6.9
“There was a long waiting list”	Social reasons	9	6.9
“I was afraid they would arrest or deport me”	Migrant reasons	7	5.3
“I didn't know any good doctor or specialist”	Personal reasons	6	4.6
“I neglected it”	Personal reasons	6	4.6
“It was too far to travel / I had no means of transportation”	Social reasons	6	4.6
“I could not get off work/childcare or other”	Social reasons	3	2.3
“I wanted to see if the problem would get better without going to the doctor”	Personal reasons	3	2.3
“I'm afraid of doctors, hospitals, exams, treatment”	Personal reasons	2	1.5
“I wanted to be examined by a doctor of a specific gender”	Personal reasons	1	0.8
Other reason	Personal reasons	20	15.3
